# *Brucella abortus* antigen omp25 vaccines: Development and targeting based on *Lactococcus lactis*

**DOI:** 10.1002/vms3.1173

**Published:** 2023-06-05

**Authors:** Somaye Tirbakhsh Gouran, Abbas Doosti, Mohammad Saeid Jami

**Affiliations:** ^1^ Department of Biology Shahrekord Branch, Islamic Azad University Shahrekord Iran; ^2^ Biotechnology Research Center Shahrekord Branch, Islamic Azad University Shahrekord Iran; ^3^ Cellular and Molecular Research Center Basic Health Sciences Research Institute, Shahrekord University of Medical Sciences Shahrekord Iran

**Keywords:** brucellosis infection, *Lactococcus lactis*, omp25 protein, cloning

## Abstract

**Background:**

Most *Brucella* infections take place on mucosal membranes. Therefore, creating vaccinations delivered through the mucosa may be crucial for managing brucellosis. Consequently, we assessed the efficacy of a recombinant oral antigen delivery system based on *Lactococcus lactis* for *Brucella abortus* omp25 antigen.

**Method:**

Oral vaccinations with *L. lactis* transformed with pNZ8148 variants encoding for omp25 (pNZ8148:omp25) and free‐pNZ8148 were administered to mice. On day 30, following immunization in animal groups, anti‐omp25‐specific IgG1 antibodies were assessed by the ELISA test. Additionally, nasal and bronchoalveolar lavages containing omp25‐specific secretory IgA (sIgA) were analysed by ELISA. ELISA test and real‐time PCR were also used to analyse cytokine responses up to 28 days following the last boost. In addition, the protective potential of *L. lactis* pNZ8148:omp25 vaccines was assessed in BALB/c mice by exposing them to the *B. abortus* strain.

**Results:**

Based on the initial screening results, the omp25 protein was identified for immunogenicity because it had the maximum solubility and flexibility and antigenic values of 0.75. The produced plasmid was digested using KpnI and XbaI. By electrophoretic isolation of the digestion fragments at 786 bp, the omp25 gene, the successful production of the recombinant plasmid, was confirmed. Antigen expression at the protein level revealed that the target group generated the 25 kDa‐sized omp25 protein, but there was no protein expression in the control group. Fourteen days after priming, there was a considerable amount of omp25‐specific IgG1 in the sera of mice vaccinated with pNZ8148–Usp45–omp25–*L. lactis* (*p* < 0.001 in target groups compared to the phosphate‐buffered saline control group). IFN‐γ and TNF‐α levels were more significant in samples from mice that had been given the pNZ8148–Usp45–omp25–*L. lactis* and IRBA vaccinations, in samples taken on days 14 and 28, respectively (*p* < 0.001). The pNZ8148–Usp45–omp25–*L. lactis* and IRBA immunization groups had significantly greater IL‐4 and IL‐10 transcription levels than the other groups. The spleen portions from the pNZ8148–Usp45–omp25–*L. lactis* and IRIBA vac group had less extensive spleen injuries, alveolar oedema, lymphocyte infiltration and morphological damage due to the inflammatory process.

**Conclusion:**

Our study offers a novel method for using the food‐grade, non‐pathogenic and noncommercial bacterium *L. lactis* as a protein cell factory to produce the novel immunogenic fusion candidate romp25. This method offers an appealing new approach to assessing the cost‐effective, safe, sustainable, simple pilot development of pharmaceutical products.

## Graphical abstract

Graphical Abstract:

- We assessed the efficacy of a recombinant oral antigen delivery system based on *Lactococcus lactis* for *Brucella abortus* omp25 antigen.
- The omp25 protein was identified for immunogenicity because it had the maximum solubility and flexibility and antigenic values of 0.75.
- Our study offers a novel method for using the food‐grade, non‐pathogenic and noncommercial bacterium *L. lactis* as a protein cell factory to produce the novel immunogenic fusion candidate romp25.

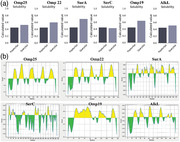

## 1 INTRODUCTION

Brucellosis is an endemic illness spread widely worldwide by domesticated animals infected with *Brucella* spp. (Franc et al., 2018). Specifically, *Brucella melitensis* and *Brucella abortus* are the major causes. The most common ways to get brucellosis are eating, breathing or being close to animal bites or sores (Franc et al., 2018; Ghajari et al., 2021). Like other naturally occurring intracellular infections, *Brucella* can exist outside of eukaryotic cells but needs to infect and proliferate inside living things to thrive (Hosseini et al., 2022). Because *Brucella* is so effectively suited to the intracellular environment, it should be referred to as a facultative extracellular intracellular parasite (Pizarro‐Cerdá et al., 2000). This bacterium causes feverish illnesses in people called ‘undulant fever’ and abortions in cattle, goats and sheep, creating a burden for both the economy and the general health problem (Jamil et al., 2021). Tolerance to *Brucella* relies on accumulated cell‐mediated immunity (CMI), just as tolerance to other facultative intracellular bacterial infections (Saxena et al., 2018). Most research on the immune reaction to *Brucella* spp. has been done on mice. The protective immunity in this mouse model appears to be controlled by both cellular and humoral effector pathways (Dorneles, Sriranganathan, et al., 2015; Dorneles, Teixeira‐Carvalho, et al., 2015).

An immunological reaction mediated by CD4+ and CD8+ T lymphocytes is particularly critical to managing the pathogen, according to in vivo evaluation. Gamma interferon‐producing CD4+ T cells within these lymphocyte subtypes are crucial for the immune system's ability to recover from infection (Reschner et al., 2008). Live attenuated variants of *B. abortus*, such as variant RB51 or variant 19, are used in the vaccination vs. brucellosis in livestock and wild ruminants. Although variant 19 effectively triggers a protective immune system response, it harms humans and causes abortion when given to pregnant cattle (Ribeiro et al., 2002). Even though variant RB51 is the preferred brucellosis vaccination for cattle is produced from the virulent *B. abortus* 2308 variant and is not, like strain 19, advised for pregnant animals (Dorneles, Sriranganathan, et al., 2015; Dorneles, Teixeira‐Carvalho, et al., 2015; Kargar et al., 2014). Additionally, as there are presently no human vaccines on the market, a different strategy to immunize humans and animals against brucellosis would be to utilize a food‐grade oral vaccine expressing a shared protein from *B. melitensis* and *B. abortus* (Sáez et al., 2012).

Alternative vaccination methods utilizing *B. abortus* proteins with known immunogenic characteristics have recently been researched. Live vaccination techniques are refined using isolated proteins generated in either life *Escherichia coli* or recombinant attenuated *B. abortus* isolates. A cell‐mediated immune response is necessary for an intracellular pathogen vaccination to be successful. Ribosomal protein L7/L12, an antigen of *B. abortus*, induces a cell‐mediated immune response and provides protective immunity in mice. Live attenuated vaccines may cause solid CMI responses, often highly effective vs. brucellosis. It prevents brucellosis in livestock by employing attenuated isolates of *B. melitensis* Rev1 and *B. abortus* S19 and RB51. These vaccine formulations are far from ideal because they have various drawbacks, such as the fact that they are harmful for human use and might cause miscarriage in pregnant calves. These vaccines are also regarded as too virulent or dangerous for animal use (Rezaei et al., 2020). Antigen injection by live lactic acid bacteria (LAB) as food‐grade, non‐invasive and non‐pathogenic vaccines is a promising immunization approach (Beiranvand et al., 2022). This strategy might eliminate possible drawbacks of using live *B. abortus* isolates as protein delivery vehicles, such as interference with diagnostic testing, residual pathogenicity and reversion hazard (Heidary et al., 2022). It offers a widespread and affordable vaccine administration method. An effective immune system response has been demonstrated following mucosal vaccination using LAB, which has been genetically engineered to generate bacterial and viral proteins (Levit et al., 2022).

These positive outcomes suggest the viability of the LAB‐based vaccination strategy. The potential to concurrently produce humoral and cell‐mediated immune function at mucosal locations and throughout the body is another benefit of mucosal‐delivered vaccinations (Taghinezhad et al., 2021). Non‐invasive, non‐pathogenic *Lactococcus lactis* is a potential antigen delivery device for mucosal vaccination inside LAB (Bermúdez‐Humarán et al., 2011; Piri‐Gharaghie, Beiranvand, et al., 2022; Piri‐Gharaghie, Doosti, et al., 2022). The digestive tract will not get colonized by *L. lactis*, even though it may survive transit through the gastrointestinal system (Daniel et al., 2013). *L. lactis* expression systems have been developed during the past 10 years due to gene engineering. Recombinant isolates of *L. lactis* have been demonstrated to generate specific immunological reactions in mice (Plavec et al., 2021). Viral, bacterial and parasite antigens have been produced in *L. lactis*. Additionally, IL‐12 (IL‐12, a cytokine aid in fighting against several bacterial, viral and parasite infections) has been made using *L. lactis* (Zeinali et al., 2022). Additionally, the treatment of *L. lactis* isolates secreting IL‐12 and *L. lactis* isolates displaying cell wall–anchored HPV‐16 E7 proteins significantly improved the immune reaction following co‐administration (Bermúdez‐Humarán et al., 2003).

The discovery of immunogenic proteins that may elicit a cell‐mediated immune response, which is necessary to fight the pathogen's intracellular location, has been the foundation of current techniques for developing novel vaccines vs. *B. abortus* (Piri‐Gharaghie, Beiranvand, et al., 2022; Piri‐Gharaghie, Doosti, et al., 2022). The omp25, the antigen of *B. abortus*, can boost the immune defence (Li et al., 2022). Mice that received immunization with pure omp25 showed exceptional protection against *B. abortus* infection (Zhang et al., 2022). This study examines the possibility of using *L. lactis* to treat mice with *B. abortus* omp25 as a vaccine.

## 2 MATERIALS AND METHODS

### 2.1 Bioinformatics analyses: screening of vaccine candidate proteins

In the identification of a possible vaccination candidate for this investigation, 35 genome sequences of *B. abortus* strains were found using the Vaxign website (http://www.violinet.org/vaxign/). Candidates for vaccines were selected from the 10 gene‐coding proteins research participants. The requirements were:

- A threshold of one transmembrane helix (≤1).
- A possibility of adhesion higher than 0.51.
- A lack of similarity to human or mouse proteins.

The vaccine candidate components must not interact with human or mouse biomolecules to reduce the likelihood of a host cell interacting with the vaccination. The proteins were examined for this purpose using the host protein *Homo sapiens*/mice and the BLASTp web service on the NCBI website. The proteins from the *B. abortus* genus will be utilized for further investigation if the BLASTp web service confirms the protein specificity. The existence of various proteins in the *B. abortus* proteome was examined using the UniProt database (http://www.ncbi.nlm.nih.gov/protein), and the NCBI saved the sequence of amino acids in FASTA format for subsequent research. To establish the exact location of proteins in the *B. abortus* bacteria, the CELLO program (http://cello.life.nctu.edu.tw/) has been used. It can identify the location of molecules outside the cell with a Localization Reliability ≥1.5 on the outer or inner membrane and determine if the protein is cytoplasmic or periplasmic. The VaxiJen software (http://www.ddg‐pharmfac.net/vaxijen/VaxiJen/VaxiJen.html) was used to calculate the antigenicity of the selected proteins, using a cut‐off of 0.6. The physicochemical characteristics of the *B. abortus* outer membrane proteins (OMPs) were examined using a bioinformatics method. All potential dominant B‐cell and T‐cell epitopes were then predicted. Optimum Antigen Design Tool, a piece of bioinformatics software, was used to anticipate the B‐cell epitopes (GenScript, China). The secondary structure, surface availability, solubility in water, flexibility and antigenic index of B‐cell epitopes were anticipated. The A and E subregions of the mouse MHC‐II genes were analysed and predicted using the Immune Epitope Database Analysis Resource (IEDB) (https://tools.iedb.org/mhci/) when determining T‐cell epitopes. As a potential vaccination candidate, the proteins with more than five B‐cell and five T‐cell epitopes were screened (Piri‐Gharaghie, Doosti, et al., 2023).

### 2.2 Animals

Female BALB/c mice (6–8‐week old) were purchased from the Biotechnology Research Animal Laboratory Center. Following the guidelines of the Ethics Committee, mice were maintained in specified pathogen‐free environments. All animal protocols were carried out following the regulations established by the Institutional Animal Care and Use Committee.

### 2.3 Positive control, bacterial strains and growth conditions

The *E. coli TOP10F* and *L. lactis IBRC‐M 11051* isolates were purchased. *L. lactis* isolates were cultivated in M17 medium (Ibrsco, Iran) at 30°C and in anaerobic condition with a 1% glucose supplement. *E. coli TOP10F* isolates were cultivated at 37°C in Luria‐Bertani media. *Brucella* isolates were cultivated for 72 h at 37°C in tryptone soy broth made by Difco Laboratories in Detroit, Michigan, USA. Biosecurity level 3 equipment was used for all live *Brucella* investigations. Brucellosis vaccine IRIBA (IRIBA Vac) was prepared as a positive control. This vaccine contains the live mass of the IRBA strain of *B. abortus* bacteria (Piri‐Gharaghie, Ghajari, et al., 2023).

### 2.4 Recombinant plasmid construction

Reverse vaccinology analysis was performed to identify vaccine antigen candidates, and *Brucella* omp25 protein (Accession number AFJ79953.1) was selected as a vaccine candidate. For the expression of the omp25 *Brucella* protein in *L. lactis*, the gene for omp25, along with its promoter, was synthesized and inserted into the pNZ8148 lactococcal expression vector by GENEray company (GENEray, China). A signal peptide sequence (31 amino acids) entitled Usp45 (accession number ABY84357) was inserted at the start of the target gene (omp25) sequence to provide a secretory characteristic. To transmit plasmid constructs into *L. lactis*, *E. coli* was initially used for transformation. The transformed strains were isolated using the matrix approach and re‐cultured at 37°C. In this study, an endonuclease‐free plasmid extraction kit was subsequently used to purify the plasmids from the *E. coli* bacterium. The plasmid was introduced into *L. lactis* by an electro‐transformation procedure (four times optimal pulse with capacity: 25 microfarads, resistance: 200 ohms, voltage: 2500 volts, time: 4–5 ms). The following antibiotic concentrations were used to select plasmids: 100 μg/mL of ampicillin for *E. coli* and 5 μg/mL of chloramphenicol for *L. lactis*.

### 2.5 Confirmation of the cloning of omp25 into the pNZ8148 vector

The PCR reaction was conducted to monitor omp25 using specific primers (Table 1). A 20‐mL PCR reaction contains 2 mL of ×10 PCR buffer (Yekta Tajhiz Azma, Iran), 2 mM MgCl_2_, 200 μM dNTPs (Yekta Tajhiz Azma, Iran), 10 pmol of each primer (CinnaGen, Iran), 100 ng of plasmid DNA and 1 unit of Taq DNA polymerase enzyme (Yekta Tajhiz Azma, Iran). The PCR temperature protocol consists of an initial annealing step at 95°C for 5 min, followed by 30 repeated stages at 94°C for 1 min, 52°C for 1 min and 72°C for 1 min. Finally, the final elongation was performed at 72°C for 5 min. The PCR product was electrophoresed on a 1% agarose gel containing ethidium bromide, and the bands were observed and recorded with a UVITech (England) gel imaging device. The recombinant vector was subjected to enzyme digestion and sequencing by GENEray company, employing the restriction enzymes KpnI and XbaI.

**TABLE 1:** Primers used in this study.

Gene	Sequence (3′ → 5′)	TM (°C)	Size (bp)
*Omp25*	F: 5′‐ATGACCTTTAAAAATTTACTTGGTG‐3′ R: 5′‐TTAAAATTTATAAGCGACACCAAG‐3′	52	693
*GAPDH*	F: 5′‐TGTGTCCGTCGTGGATCTGA‐3′ R: 5′‐CCTGCTTCACCACCTTCTTGA‐3′	60	78
*IFN‐γ*	F: 5′‐AGCGGCTGACTGAACTCAGATTGTAG‐3′ F: 5′‐GTCACAGTTTTCAGCTGTATAGGG‐3′	60	199
*IL‐10*	F: 5′‐CTTGGGACTGATGCTGGTGAC‐3′ R: 5′‐TCTTTTCTCATTTCCACGATTTC‐3′	60	162
*IL‐4*	F: 5′‐CGAAGAACACCACAGAGAGTGAGCT‐3′ R: 5′‐GACTCATTCATGGTGCAGCTTATCG‐3′	60	180
*TNF‐α*	F: 5′‐TCCTTGGCAAAACTGCACCT‐3′ R: 5′‐TCCTTGGCAAAACTGCACCT‐3′	60	183

### 2.6 Expression and immunodetection of omp25

The recombinant *L. lactis* organism carrying pNZ8148:omp25 was used to culture fresh medium overnight at a dilution of 1/100 to induce the nisin promoter. The colonies were cultured for 1 h at an optical density of 600 nm (OD600) of 0.6 (∼0.6) before cell fractionation and protein identification. Nisin‐induced *L. lactis* pNZ8148:omp25 was obtained by centrifuging at 12,000 × *g* for 10 min at 4°C, concentrating the bacterial protein supernatants by 50 times relative to their original volume, and then analysing it using 12% sodium dodecyl sulphate–polyacrylamide gel electrophoresis before electrotransferred it onto the nitrocellulose membrane. The membranes were treated with a mouse IgG monoclonal antibody anti‐omp25 (Rozhan AZMA, Iran) diluted 1:500 on phosphate‐buffered saline (PBS) for 60 min at 37°C, after blocking with TBST (tris‐buffered saline, 0.05% Tween‐20) solution containing 5% skimmed milk overnight at 4°C. The samples were washed, followed by 60‐min incubation with a 1:2000 dilution of HRP‐conjugated rabbit anti‐mouse IgG from Rozhan AZMA, Inc., Iran. Using diaminobenzidine (Merck), as recommended by the manufacturer, binding was visualized.

### 2.7 Grouping of animals and schedule for oral immunization

In this investigation, 120 female BALB/c mice aged 6–8 weeks and weighing 15–20 g were employed. Animals have injected pNZ8148–omp25 through oral immunization (100 μg of pDNA). A total of 80 mice were split into 5 groups of 20, whereas an additional 20 mice served as negative controls (*n* = 20) and received PBS. The quantity of mice used in each group is shown in Table 2. The control and recombinant strains of *L. lactis* pNZ8148:omp25 were cultivated as described earlier and stimulated for 1 h with nisin. An oral pipette was used to administer 10^8^ *L. lactis* pNZ8148:omp25 colony forming unit (CFU) three times to a group of 20 mice. The treatment was given over 3 days (days 0–2, 14–16 and 28–30), separated by 15 days. The animals in the control groups received 10^8^ *L. lactis* or PBS vaccinations. Another group of mice received a subcutaneous injection vaccination with 2 × 10^8^ CFU of the IRIBA vaccine (Razi, Iran). PBS of 200 μL was vacuum‐cleaned at zero time as a positive control.

**TABLE 2:** The number of mice used in this experiment.

Group number	Injection composition	Number of mice	Average weight of mice	Type of injection	Time of injection	Injection day
1	pNZ8148–Usp45–omp25–*Lactococcus lactis*	20	19.7 ± 0.5	Oral	Every 8 h for 3 days	0–2, 14–16, 28–30
2	pNZ8148–*L. lactis*	20	18.2 ± 0.7	Oral	Every 8 h for 3 days	0–2, 14–16, 28–30
3	*L. lactis*	20	18.2 ± 0.7	Oral	Every 8 h for 3 days	0–2, 14–16, 28–30
4	IRIBA Vac	20	17.9 ± 1.02	Subcutaneous injection	Every 8 h for 3 days	1, 14, 28
5	pNZ8148	20	18.1 ± 1.22	Oral	Every 8 h for 3 days	0–2, 14–16, 28–30
6	PBS	20	19.4 ± 0.68	Oral	Every 8 h for 3 days	0–2, 14–16, 28–30

Abbreviations: omp25, outer membrane protein 25; PBS, phosphate‐buffered saline.

### 2.8 ELISA analysis

Two days before each inoculation and 15 days after the final vaccination, mice were bled, and serum samples were collected (five mice per group). The substance was gathered and kept at −70°C. By using an indirect ELISA following a predetermined procedure, the presence of sera G1 (IgG1) and secretory IgA (sIgA) from nasal lavages with specificity to omp25 was identified. Purified romp25 was collected, diluted to 2 μg/mL in carbonate–bicarbonate buffer (pH 9.6) and utilized to coat the wells of a polystyrene plate with crude *Brucella* proteins, an extract acquired from organisms treated with a hyperosmotic salt solution and sonication, at a concentration of 10 μg/mL within the same buffer (Nunc‐Immuno plate with MaxiSorp surface). Isotype‐specific goat anti‐mouse horseradish omp25 conjugates were administered at a dilution of 1:1000. The average specific OD450 for 10 sera from non‐immunized animals evaluated at a concentration of 1:50 was multiplied by the standard deviation to get the assay's cut‐off ratio. The data were normalized and represented as the endpoint titre OD of antigen‐specific sIgA concerning 1 μg of total IgA in the sample to account for differences in the effectiveness of recovering secretory antibodies among individuals. Total IgG1 values are shown as the mean OD450 at a plasma dilution of 1:100.

### 2.9 Detection of cellular immune response

1. **ELISA assay**: The levels of IFN‐γ, TNF‐α, IL‐10 and IL‐4 were measured to evaluate the cellular immune response caused by the oral vaccination. According to the owner's manual, the amounts of functional and proinflammatory cytokines were determined in the serum sample using karmania pars gene ELISA kits (KPG, Iran). The coating antibody was coated overnight at 4°C onto a 96‐well plate (0.2 μg/well). PBS with 0.5% BSA and 0.1% Tween 20 was then used to block the plate. Then, 100 μL of the sample was distributed into two replicated wells, and 50 μL of 1 μg/mL detecting antibody was added. The plate was sealed and shaken for 2 h at room temperature (700 rpm). Following washes with PBS containing 0.1% Tween 20, 100 μL of streptavidin–HRP (1:1024) was added and incubated for 30 min on a shaker at room temperature (700 rpm).
2. **Real‐time PCR assay**: The spleen was extracted under aseptic circumstances and stored in liquid nitrogen at −198°C after the mice were anaesthetized. Next, RNA extraction and cDNA synthesis from spleen tissue was performed according to the YTA kits’ instructions (Yekta Tajhiz, Iran). Real‐time PCR was performed with the YTA SYBR Green master mix (Yekta Tajhiz, Iran), and the GAPDH gene was used as an internal control. Fifteen microliters of reaction volume consisting of 0.5 μL cDNA, 0.5 μL forward primer, 0.5 μL rivers primer, 10 μL master mix and 3.5 μL of double sterile distillation water were used for real‐time PCR. The temperature cycle program also included initial denaturation at 95°C for 10 min, followed by 40 cycles at 95°C for 20 s and 60°C for 40 s. The relative gene expression of IFN‐γ, TNF‐α, IL‐10 and IL‐4 were calculated by using the 2 − ΔΔ*Ct* method and normalized to GAPDH levels in each sample.

### 2.10 Challenge experiments

The protection experiments were carried out following Piri‐Gharaghie et al. A total of 10 mice from each group were exposed to 10^4^ CFU of *B. abortus* strain 2308 intraperitoneally 2 weeks after the previous dose. The mice were tracked for 7 days, and each group's body weight, clinical score and survival rate were recorded every day. Each mouse's overall clinical sign was rated on a range of 0 to −5 on a sliding scale. Individual clinical ratings ranged from 0 (normal, active, healthy), −1 (slightly sick, slightly ruffled fur, otherwise normal), −2 (ill, ruffled fur, sluggish movement, hunching), −3 (extremely sick, ruffled hair, prolonged movement, stooped, eyes shut), −4 (moribund) and −5 (dead). The infected mice were slaughtered according to ARRIVE recommendations (PLOS Bio 8(6), e1000412, 2010) 2 weeks later, and the spleens were removed under aseptic circumstances, homogenized and plated in Blood Agar (BIO3P, Iran) to count the number of *B. abortus* CFU per organ. The average log10 CFU for the treatment group was subtracted from the median log10 CFU for the standard matching control to get log10 units of immunity.

### 2.11 Histopathological examination

Livers were extracted aseptically and fixed in 10% formalin. After embedding in paraffin, slices were examined histopathologically under a microscope after staining with haematoxylin and eosin. The proportion of the lesion area among the whole liver area was used to assess liver damage using an ImagePro macro.

### 2.12 Statistical analyses

GraphPad Prism 5.0 was used to examine the data and perform statistical tests. A one‐way analysis of variance was used to compare means, followed by a Tukey–Kramer post hoc test with a 95% confidence interval. A Chi‐square test with Yates’ correction was used to compare the survival rates between immunized mice and the control group. Differences were considered significant at *p* < 0.05 and *p* < 0.01.

## 3 RESULTS

### 3.1 A potential vaccine candidate has been discovered

**FIGURE 1:**
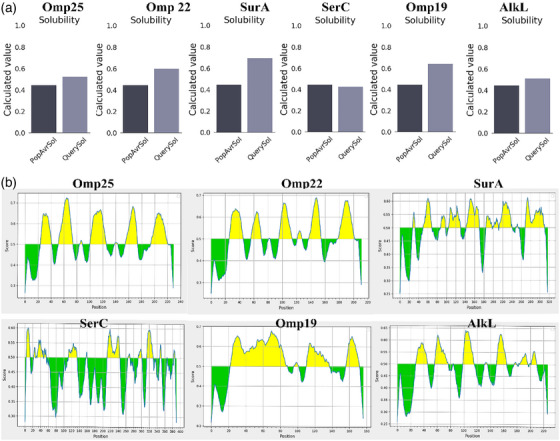
(A) The solubility of the screened proteins according to the results of the Protein‐Sol online site; (B) the number of epitopes of each of the screened proteins according to the results of the Immune Epitope Database (IEDB) site (https://tools.iedb.org/mhci/).

The Vaxign database identified 44 proteins with top qualities. Then, 18 hypothetically pathogenic proteins were selected. The potential vaccine candidates were reduced to 6 after protein antigenicity was evaluated. After the UniProt dataset confirmed the existence of six proteins in *B. abortus*, the access number of these proteins was chosen on the NCBI website. They were categorized as extracellular, outer membrane, periplasmic, inner membrane and cytoplasmic proteins based on the output of the CELLO web application. Most proteins selected for *B. abortus* were confirmed for their specificity by BLASTp protein results, lowering the possibility of host cell interaction with the DNA vaccine. The results of the preliminary screening of six *B. abortus* antigens are shown in Table 3. Based on the initial screening results, the omp25 protein was identified for immunogenicity because it had the maximum solubility and flexibility and antigenic values of 0.75. Based on the screening results, the omp25 protein was identified as immunogenic because it has maximum solubility, which helps the protein reactivity with immune cells. Moreover, omp25 is more flexible than other proteins. This leads to immune cells recognizing different epitopes of this protein in different spatial structures. On the other hand, omp25 had higher antigen values (0.75) than other proteins, which indicates that it has a high potential to activate immune cells. The prospective vaccine candidate omp25 showed B‐cell linear epitopes with a value of 0.5 that are disseminated with the proteins, according to IEDB research. Omp25 had five B‐cell epitopes and five T‐cell epitopes. The quantity of surface‐exposed conformational B‐cell epitopes is shown in Figure 1.

**TABLE 3:** Screening of 6 *Brucella abortus* proteins based on reverse vaccinology.

	Accession number	CELLO analysis		Protein‐Sol			IEDB	
Name		Localization	Reliability	Hydrophilicity		Hydrophilicity	Flexibility	VaxiJen
Omp25	AFJ79953.1	Extracellular	3.93^a^	p*I*	9.60	0.79	0.85	0.75
		Cytoplasmic	1.28^a^	Solubility	0.522			
Omp22	AAS84601.1	Extracellular	1.97^a^	p*I*	9.48	0.67	0.77	0.74
		Outer membrane	1.53^a^	Solubility	0.598			
AlkL	SUW22850.1	Extracellular	2.53^a^	p*I*	6.41	0.65	0.68	0.69
		Outer membrane	0.84	Solubility	0.511			
Omp19	CDL77301.1	Outer membrane	2.71^a^	p*I*	9.93	0.48	0.61	0.65
		Extracellular	2.20^a^	Solubility	0.644			
serC	SUW22956.1	Outer membrane	4.79^a^	p*I*	5.94	0.48	0.58	0.35
		Periplasmic	0.09	Solubility	0.427			
SurA	SUW22048.1	Periplasmic	2.68^a^	p*I*	10.35	0.45	0.57	0.47
		Extracellular	1.44^a^	Solubility	0.696			

Abbreviation: IEDB, Immune Epitope Database.

^a^ Significance level >1.2.

### 3.2 Generating and identifying recombinant pNZ8148–Usp45–omp25–*L. lactis*

**FIGURE 2:**
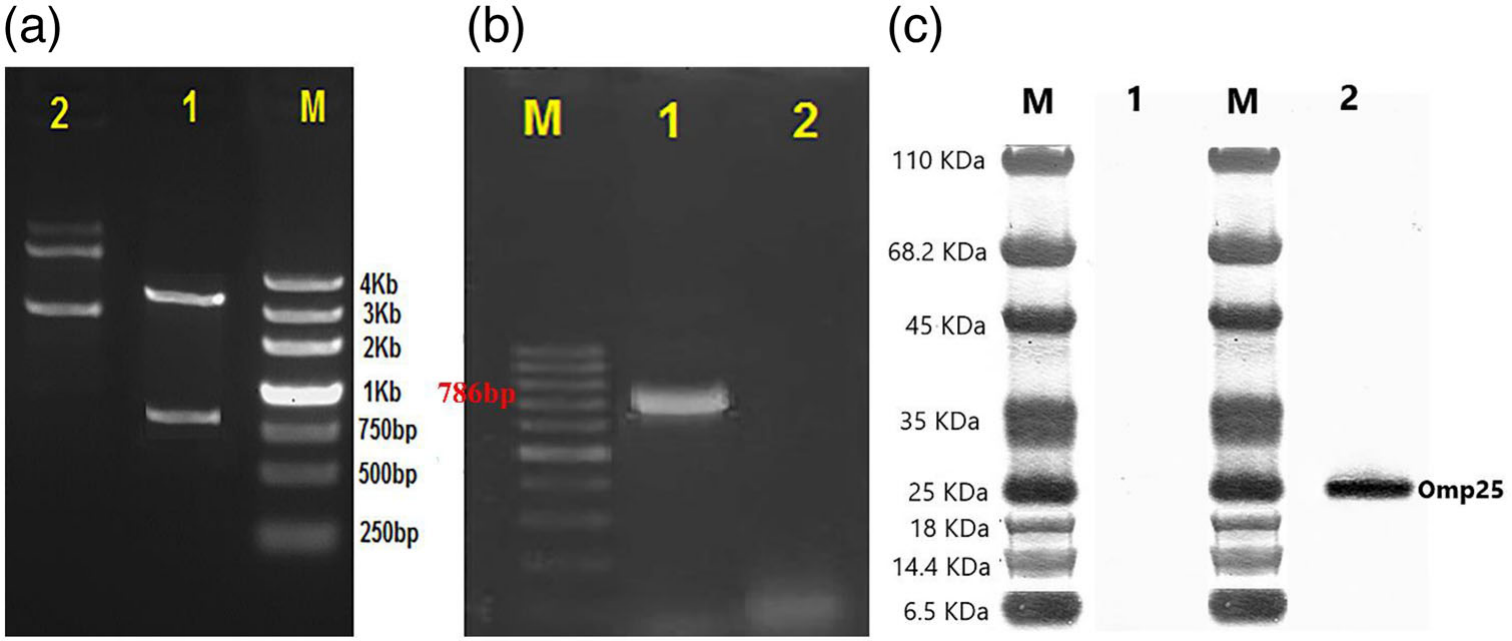
(A) Lane 1: double enzyme digestion shows the bp786 band of the gene along with the signal peptide (693 + 93 bp), lane 2: recombinant plasmid before enzymatic digestion; (B) line 1: expression of omp25 gene transcription at the mRNA level and the formation of a 786 bp band in *Lactococcus lactis* transformed with a recombinant vector, line 2: *L. lactis* transformed with a vector lacking the target gene; (C) verify the presence of recombinant protein in transformed *L. lactis* bacteria using a Western blot. Line 1: *L. lactis* bacteria transformed with a vector lacking the target gene, M: marker, line 2: *L. lactis* bacteria transformed with the recombinant vector pNZ8148–Usp45–omp25.

The DNA vaccine construct was constructed by inserting the omp25 gene into the expression vector pNZ8148. The Genetic code of the recombinant plasmid served as a measure of the cloning effectiveness. Additionally, DNA analysis showed that the recombinant plasmid's virulence gene sequence was 100% identical to that of the *B. abortus* bacterium. The produced plasmid was digested using KpnI and XbaI. By the electrophoretic isolation of the digestion fragments at 786 bp, the omp25 gene, the successful production of the recombinant plasmid was confirmed (Figure 2A). The results of the sequencing of recombinant plasmids were examined using Blast. Blast findings revealed that recombinant plasmids with an *E*‐value of 8e − 94, query cover 100% and Per. Ident was 99.86 similar to the target bacterium.

### 3.3 Expression of recombinant pNZ8148–Usp45–omp25–*L. lactis* in mRNA and secretion of omp25 in *L. lactis*

The relative amounts of mRNA expression of the omp25 Gene delivery were determined using reverse transcriptase‐PCR. The 786 bp band on the agarose gel shows that the transcription of the gene has taken place (Figure 2B). The Western blot analysis by Rojan Azma also revealed that the specific proteins were generated at the protein level. Antigen expression at the protein level revealed that the target group generated the 25 kDa‐sized omp25 protein, but there was no protein expression in the control group (Figure 2C). The electrophoresed *L. lactis* protein samples from our recombinant bacterium pNZ8148–Usp45–omp25–*L. lactis* and the bacterium transformed with an empty vector (pNZ8148) are shown in Figure 2C. The recombinant strain secreted a characteristic protein band that matches the anticipated size of the omp25 (25 kDa) upon Coomassie blue gel staining in the cell‐free media (indicated with an arrow in Figure 2C, lane 2). In isolates from the cell‐free supernatants of the recombinant bacterium pNZ8148–Usp45–omp25–*L. lactis*, Western blot analysis showed distinct single bands that were the predicted size for omp25. Cultures from *L. lactis* transformed with the empty vector pNZ8148, which lacks the romp25‐coding gene, do not contain this specific protein band (Figure 2C, lane 1). This outcome indicates that omp25 secretion can be achieved in *L. lactis* effectively.

### 3.4 Immune responses induced by oral immunization

**FIGURE 3:**
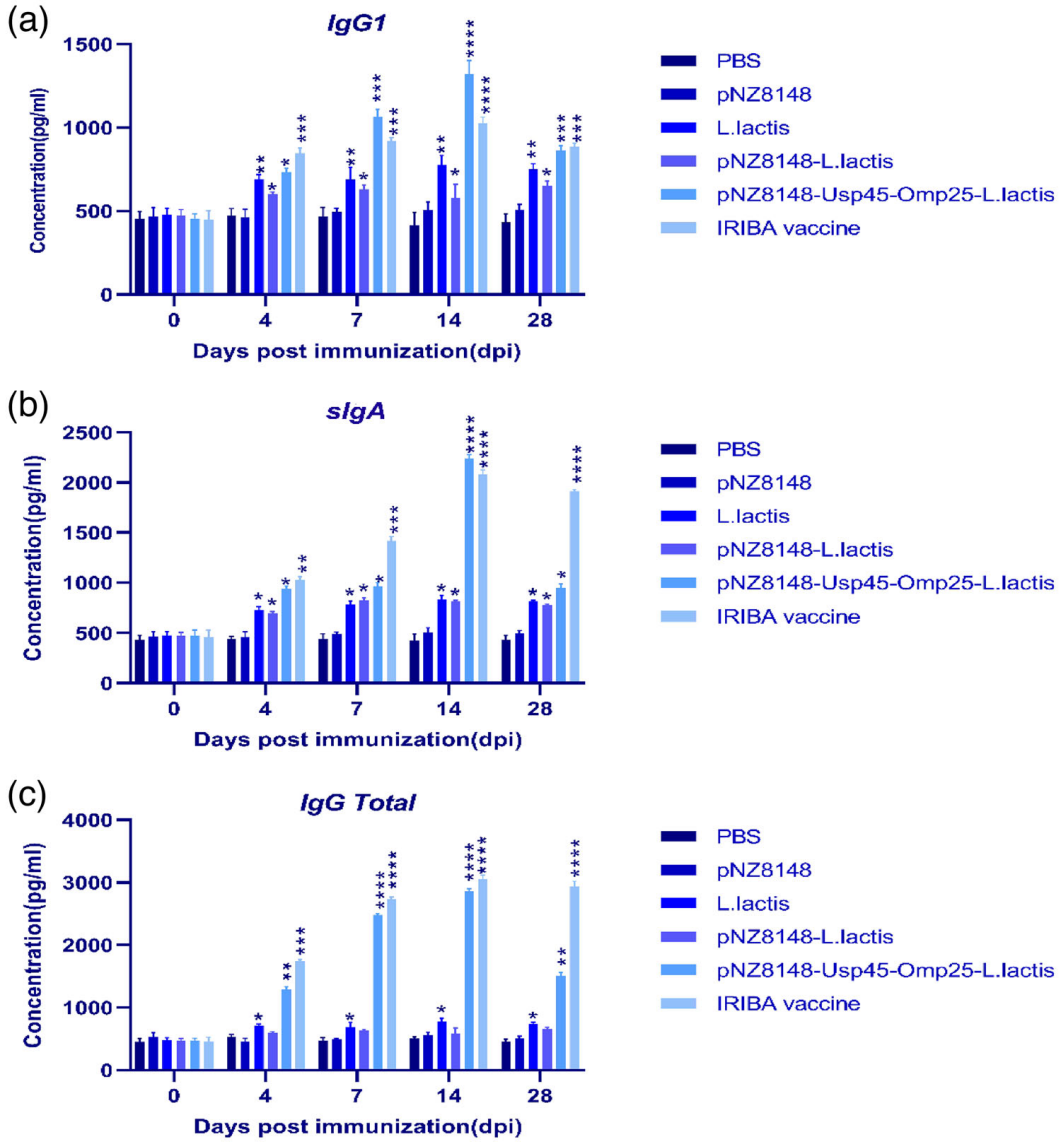
(A) Thirty days following the last vaccination, BALB/c mice exhibit humoral immunological response. 1:200 serum dilutions of romp25‐specific IgG1 from various groups of mice; (B) omp25‐specific mucosal secretory IgA (sIgA) antibodies in nasal lavage; (C) omp25‐specific mucosal total IgG antibodies in mice. Sample obtained from mice after oral immunization with different groups of vaccines. *p < 0.05, **p < 0.01, ***p < 0.001.

A prospective vaccination method involved employing non‐pathogenic LAB (*L. lactis*) as target delivery vehicles to induce humoral and cell‐mediated immune responses throughout the body, including mucosal sites. Thus, we chose to evaluate whether vaccination with *L. lactis*, which has been genetically engineered to release omp25, can induce a particular immune response in various groups, as shown in Table 2. After oral immunization, BALB/c mice were tested for omp25‐specific IgG1 or sIgA using an indirect ELISA. As seen in Figure 3A, 14 days after priming, there was a considerable amount of omp25‐specific IgG1 in the sera of mice vaccinated with pNZ8148–Usp45–omp25–*L. lactis* (*p* < 0.001 in target groups compared to the PBS control group). Similar substantial values were seen at day 28, although the levels returned to baseline compared to the PBS negative control groups. Compared to the values from mice in the PBS control group, the findings revealed 14 days after vaccination with pNZ8148–Usp45–omp25–*L. lactis*, levels of omp25‐specific IgG1 were significantly (Figure 3A; *p* < 0.0001) higher in both pNZ8148–Usp45–omp25–*L. lactis* and IRBA vaccine (positive control) groups. Even while the levels of IgG1 decreased on day 28 compared to controls, they were still considerably greater. The levels of total IgG in sera from animals that had received the pNZ8148–Usp45–omp25–*L. lactis* and IRBA vaccines were also higher than those from the negative control groups in samples obtained on days 14 and 28, respectively (*p* < 0.001) (Figure 3C). At day 28, the levels of total IgG in the pNZ8148–Usp45–omp25–*L. lactis* and IRBA vaccination groups were remarkably comparable, although they were still considerably more significant when compared to the control groups (*p* < 0.001).

We also assessed the induction of mucosal reactions in animals that had received vaccinations. Omp25‐specific sIgA titres were measured for this purpose using bronchoalveolar lavages and nasal swabs (Figure 3B). According to the findings, vaccinated mice produced more antigen‐specific mucosal IgA than PBS‐treated control mice or other groups (*p* < 0.05). Interestingly, compared to animals inoculated with control groups, mice were vaccinated with pNZ8148–Usp45–omp25–*L. lactis* displayed substantially higher sIgA titres in nasal lavages (*p* < 0.05). In contrast, IgA titres in the pNZ8148–Usp45–omp25–*L. lactis* and IRBA vaccination groups were comparable and considerably higher than those in the PBS control group (*p* < 0.05).

### 3.5 Cytokine secretion

**FIGURE 4:**
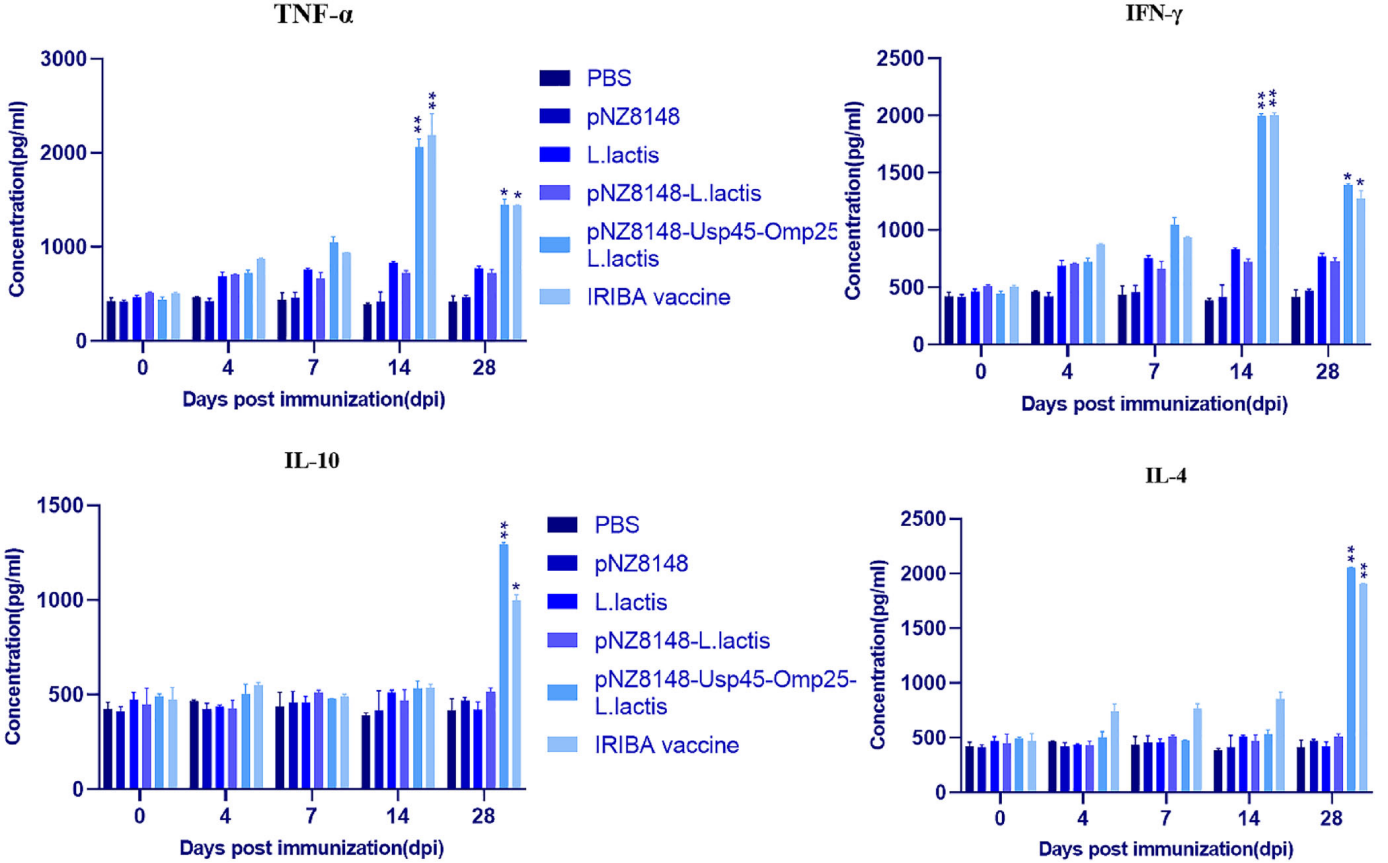
The splenocyte supernatants of the control and vaccinated groups included cytokine levels. In mice inoculated with various vaccinations, the levels of inhibitory cytokines (IL‐4, IL‐10) and functional cytokines (IFN‐γ, TNF‐α) were measured in the spleen. Phosphate‐buffered saline (PBS) served as a standard. *p < 0.05, **p < 0.01.

After re‐stimulation, cytokine production from spleen cells was examined in vitro. When challenged, splenocytes from immunized and non‐immunized mice were produced and grown with various antigens (Table 2). Unstimulated cells from animals administered PBS, pNZ8148, *L. lactis* and pNZ8148‐*L. lactis* showed no significant changes (*p* > 0.05). On the other hand, unimmunized (saline inoculation) mouse cells failed to substantially boost their IFN‐γ and TNF‐α production with re‐stimulation (*p* > 0.05). After 14 days, cells from mice were vaccinated with pNZ8148–Usp45–omp25–*L. lactis*, and IRBA secreted more IFN‐γ and TNF‐α (Figure 4). IFN‐γ and TNF‐α levels were more significant in samples from mice that had been given the pNZ8148–Usp45–omp25–*L. lactis* and IRBA vaccinations, as well as the negative control groups in samples taken on days 14 and 28, respectively (*p* < 0.001) (Figure 4). Compared to the control groups, the concentrations of IFN‐γ and TNF‐α in the pNZ8148–Usp45–omp25–*L. lactis* and IRBA vaccination groups at day 28 were impressively identical (*p* < 0.001). IFN‐γ and TNF‐α concentrations were still significantly higher on day 28 despite a decline compared to controls. IL‐4 and IL‐10 inductions in mice that had received vaccines were also evaluated. The results showed that at 14 days after vaccination, none of the mouse groups that had received the vaccine generated IL‐4 or IL‐10. Furthermore, mice were given the pNZ8148–Usp45–omp25–*L. lactis* vaccine, and the IRBA vaccination showed significantly higher IL‐4 and IL‐10 titres at 28 days after immunization (*p* < 0.05) than were mice infected with control groups. In fact, pNZ8148–Usp45–omp25–*L. lactis* and IRBA vaccination groups’ IL‐4 and IL‐10 titres were equivalent to and significantly higher than those in the PBS control group after 28 days (*p* < 0.05).

Additionally, a quantitative real‐time PCR assay was used to determine how much cytokine transcription was occurring in the spleen and small intestine. The amounts of IFN‐γ and TNF‐α transcriptions in the pNZ8148–Usp45–omp25–*L. lactis* and IRBA vaccination groups were substantially different from those in other groups. The outcomes were the same in the spleen and small intestine. The pNZ8148–Usp45–omp25–*L. lactis* and IRBA vaccination groups had significantly greater IFN‐γ and TNF‐α transcription levels than other groups. The amount of IFN‐γ and TNF‐α gene activation in the pNZ8148–Usp45–omp25–*L. lactis* and the IRBA vaccination group was considerably different from other groups’ real‐time PCR results were likewise compatible with ELISA.

**FIGURE 5:**
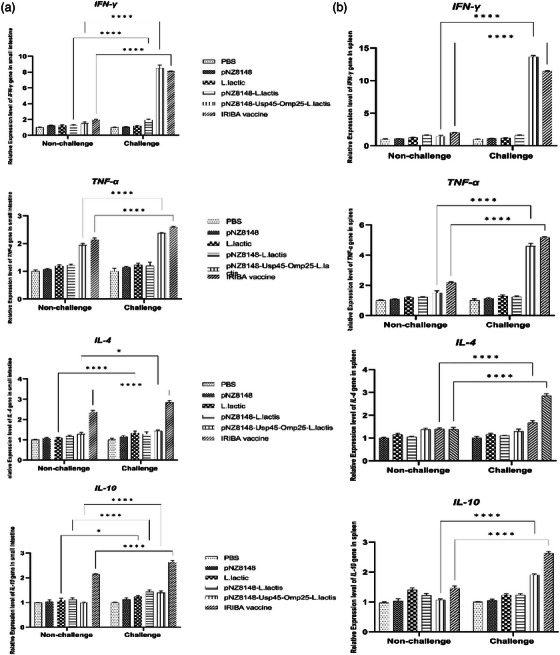
(A) Cytokine levels were found in the small intestine of the control and immunized groups; (B) cytokine levels were found in the spleen of the control and immunized groups. *p < 0.05, **p < 0.01, ***p < 0.001, ****p < 0.0001.

The pNZ8148–Usp45–omp25–*L. lactis* and IRBA immunization groups had significantly greater IL‐4 and IL‐10 transcription levels than the other groups. The results in the small intestine and spleen were identical. IL‐4 and IL‐10 transcription concentrations were considerably higher in the pNZ8148–Usp45–omp25–*L. lactis* and IRBA vaccination groups compared to other groups (Figure 5).

### 3.6 Protective activity

Using virulent *B. abortus* strains to challenge the mice, the possible protective action brought on by various vaccination groups was assessed. Three separate replicates of the experiment were run using the *B. abortus* strains. When compared to groups immunized with Ethe pNZ8148–Usp45–omp25–*L. lactis* and IRBA vaccines, the spleen weights of mice given various control vaccinations at 15 days after infection significantly decreased (Table 4). Mice were given the pNZ8148–Usp45–omp25–*L. lactis* and IRBA immunization groups showed no statistically significant changes (*p* > 0.05). The non‐challenge groups, pNZ8148–Usp45–omp25–*L. lactis* and IRBA vaccination groups did not vary significantly (*p* > 0.05) from each other. Six mice exposed to *B. abortus* were randomly selected, and their mortality, weight changes and health status were monitored daily for 15 days. According to Table 4, all control group mice died 7 days following the test. After being challenged with a fatal dose of *B. abortus* isolates, the 15‐day survival rates of mouse model vaccinated with the pNZ8148–Usp45–omp25–*L. lactis* were 87.5%, respectively. These numbers were noticeably higher than those of mice immunized with IRBA (50%). Each group's body mass and clinical symptom ratings dropped to their lowest levels 7 days after the challenge. The mice's body weight returned to normal 15 days after the experiment, and the symptoms disappeared.

**TABLE 4:** Survival percentage and clinical indicators of each group.

Group	Before challenge			After challenge			
	Body weight (g)	Clinical score	Spleen weights (mg)	Body weight (g)	Clinical score	Spleen weights (mg)	Survival rate (%)
PBS	18.3	0	398	15.75	−5	764	0
pNZ8148	18.7	0	379	15.67	−4	749	0
*Lactococcus lactis*	18.6	0	394	15.87	−3	754	12.5
pNZ8148–*L. lactis*	18.3	0	389	16.93	−1	768	12.5
IRIBA Vac	18.5	0	374	15.83	0	413	50
pNZ8148–Usp45–omp25–*L. lactis*	18.7	0	369	16.94	0	388	87.5

*Note*: 0 (normal, active, healthy), −1 (slightly sick, slightly ruffled fur, otherwise normal), −2 (ill, ruffled fur, sluggish movement, hunching), −3 (extremely sick, ruffled hair, very slow movement, stooped, eyes shut), −4 (moribund) and −5 (dead).

### 3.7 Bacterial loads and pathological changes

**FIGURE 6:**
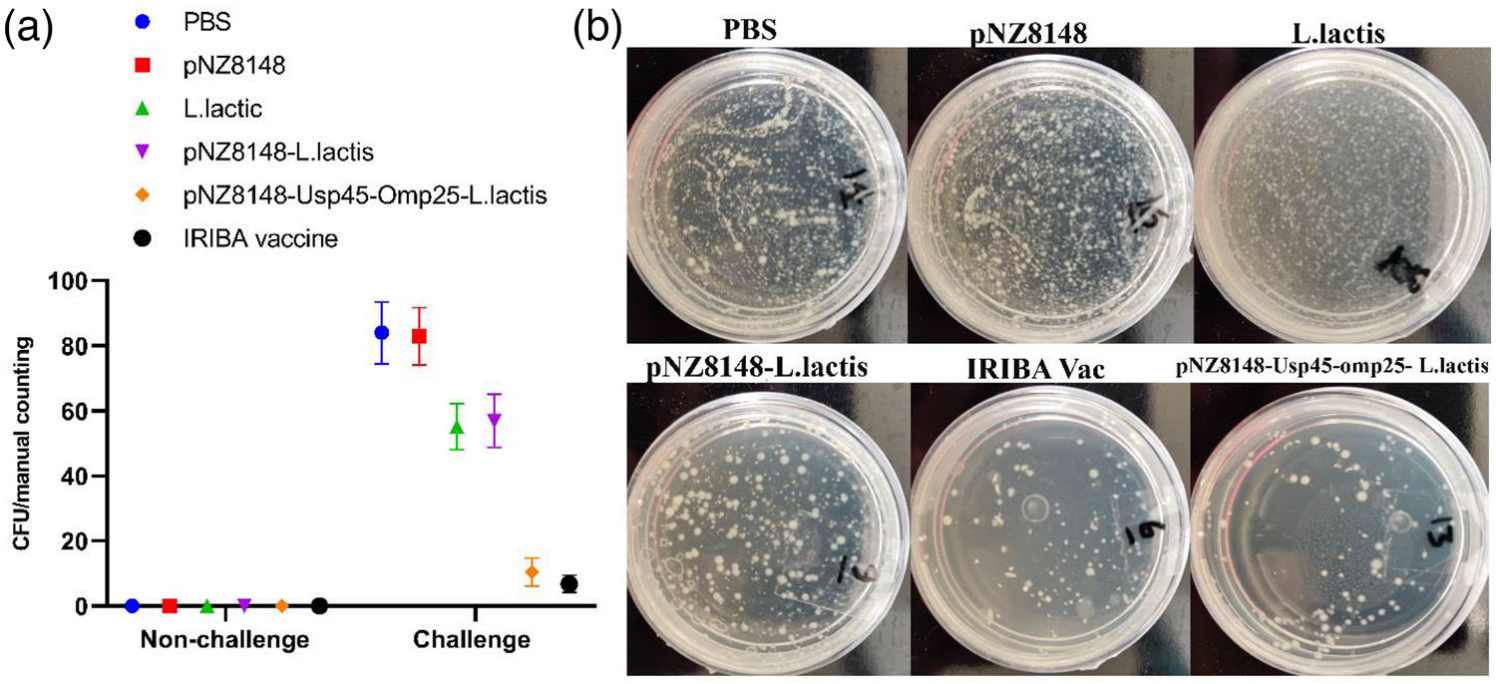
Bacterial burdens in the spleen tissues of BALB/c mice: (A) bacterial loads in the spleen of mice at 48 h post‐challenge; (B) plate agar of bacterial burdens in spleen.

**FIGURE 7:**
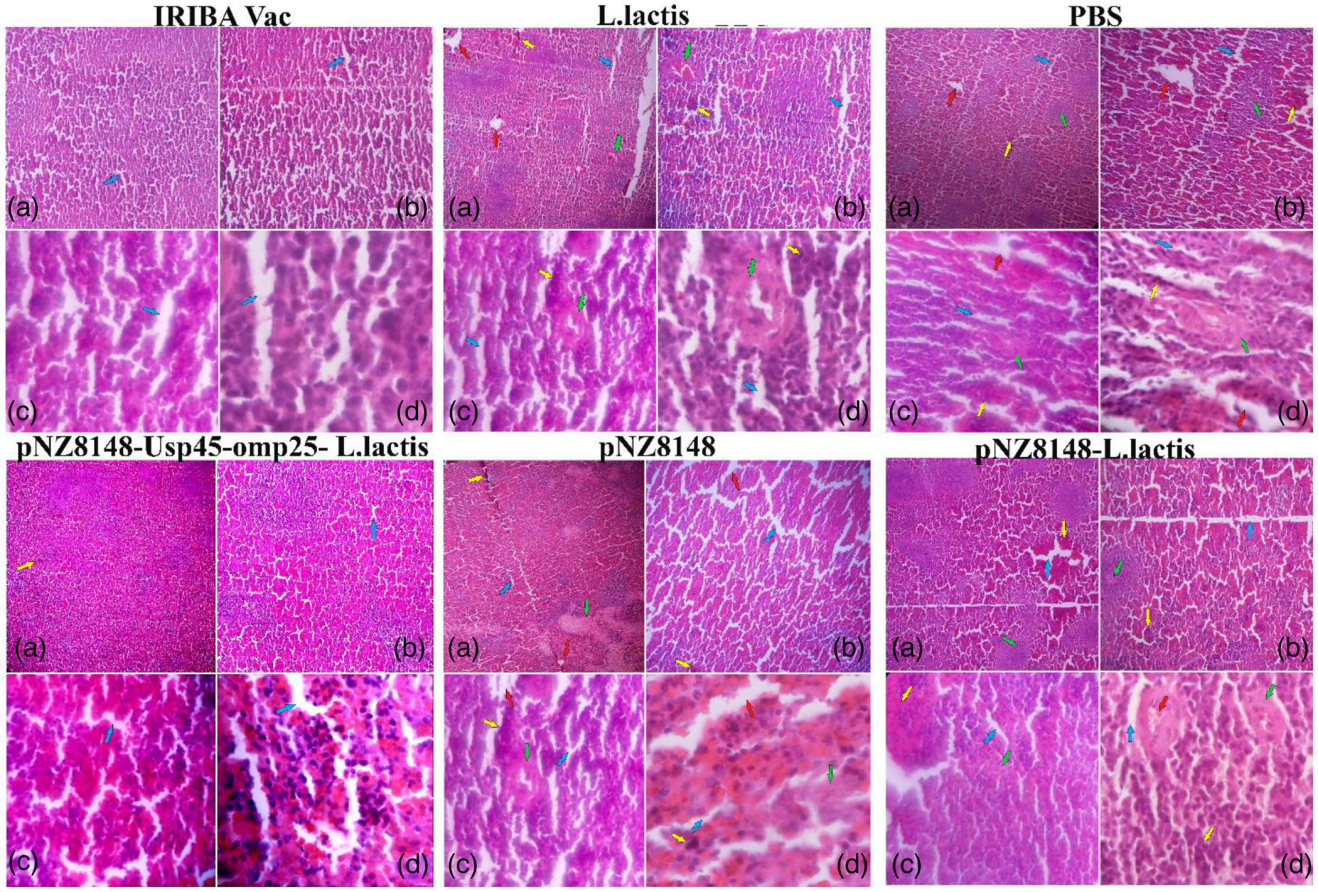
Haematoxylin–eosin staining of spleen tissue sections: (a–d), respectively, magnification ×4, ×10, ×40, ×100 – increased space of sinusoids (blue arrow), spleen tissue necrosis (green arrow), bleeding in the tissue (yellow arrow) and abscess in the tissue, brucellosis (red arrow).

Using a challenging experiment in BALB/c mice to gauge protection, we examined the effectiveness of the pNZ8148–Usp45–omp25–*L. lactis* immunization. All groups received three clinical *B. abortus* strain 2308 isolates. Six mice from each group were randomly selected to count the number of bacteria in the spleen tissue (Figure 6A). Six mice from each group were taken 48 h after the challenge, serially diluted, and then plated on *Brucella* agar with 5% blood plates. The plates were next overnight incubated at 37°C. The log10 CFU/mL value was calculated and compared following the CFU count. The spleen bacterial burdens were lower in the mice who got the pNZ8148–Usp45–omp25–*L. lactis* vaccine than they were in the animals in the other groups (Figure 6B). The challenge was followed by aseptic removal of the left spleen tissue, preservation in a 4% formalin solution, haematoxylin–eosin staining and microscopic examination. Figure 7 displays that compared to other groups, the spleen portions from the pNZ8148–Usp45–omp25–*L. lactis* and IRIBA vac groups had less extensive spleen injuries, alveolar oedema, lymphocyte infiltration and morphological damage due to the inflammatory process. The pNZ8148–Usp45–omp25–*L. lactis* vaccine groups were more typical than the other groups, with cleaner alveoli. The amount of spleen failure in the pNZ8148–Usp45–omp25–*L. lactis* groups after mice were infected with the vaccine were considerably less severe than in the other groups, suggesting that the mice had less of an inflammatory response to spleen failure.

## 4 DISCUSSION

The high incidence of brucellosis as an economically significant human illness has prompted research into various tactics, such as creating subunit vaccines and using bacterial/viral vectors, to create more efficient vaccines (Dadar et al., 2021). To overcome the drawbacks of the currently utilized live vaccines, including attenuated pathogens, safer, more affordable and simpler to deliver vaccinations are still required. *E. coli* is the most often utilized microbial host for the generation of protein. The majority of investigations utilized the *E. coli* BL21 host expression system to produce *Brucella* antigens such as omp10 and omp19, omp25, HSP and TF Proteins (Rezaei et al., 2020). However, an *E. coli* expression system does have certain limitations. In general, intracellular expression is one of the most widely utilized manufacturing techniques, although it is still expensive for industrial production. Pharmaceutical interests demand downstream purification procedures to remove endotoxin or harmful pyrogens from cell walls during manufacture (Singh et al., 2017). It has been demonstrated that *L. lactis*, a gram‐positive lactic acid microorganism with a GRAS (Generally Regarded As Safe) status, has been utilized in food production for thousands of years (Díaz‐Dinamarca et al., 2022). However, it is now used as a cell factory for producing heterologous proteins for therapeutic and commercial purposes and an intriguing alternative method for large‐scale protein production (Jawan et al., 2021).

Many advantageous health qualities of *L. lactis* may be present. According to Le Loir et al. (2005), this food‐grade bacterium does not manufacture LPS, dose *E. coli*, form inclusion bodies, sporulate or move around much (Le Loir et al., 2005). It only has one membrane, which enables the investigation of the protein's function in entire cells. A disadvantage of using live LAB mucosal vaccines is the possibility of introducing genetically altered organisms with antibiotic resistance genes into the environment and host microflora. As a result, auxotrophic mutants and food‐grade selection markers (such as bacteriocin resistance and generation), depending on the existence of these selectable markers on the plasmid of interest, could allay worries about the risk of unchecked release of nucleic acids into the environment by reducing the use of antibiotic resistance markers and harmful substances and potentially improve the applicability on an industrial level or in food products (He et al., 2012).

Additionally, *L. lactis* is a live delivery vehicle technology with several advantages over constitutive approaches, such as not being a commensal bacteria and not colonizing. Due to its immunomodulatory properties, ability to survive passage through the gastrointestinal tract, ease of uptake by M cells inducing potential immune reaction and single extracellular housekeeping protease, HrtA results in a very low rate of protein degradation, in vivo administration via oral administration and safer vaccination (Guillot et al., 2016). Only a few prior studies have examined the development and targeting of specific intracellular *Brucella* proteins in *L. lactis*, including L7/L12, GroEL heat‐shock antigen and Cu, Zn superoxide dismutase (Sáez et al., 2012). Some brucellosis vaccines focus on discovering immunodominant proteins dependent on OMPs that can trigger a strong immune reaction. Due to its superior antigenicity, availability and surface‐exposed loops predicted by thorough bioinformatics research to develop an effective vaccine in future research against brucellosis, the omp25 protein, one of the minor 25‐kDa OMPs in *Brucella* spp., was chosen as an immunogenic candidate in the current study (Piri‐Gharaghie, Beiranvand, et al., 2022; Piri‐Gharaghie, Doosti, et al., 2022).

The omp25 protein we chose for our investigation stands out because, as a part of the exoproteome of the *Brucella* cell, it is surface‐exposed, accessible to monoclonal antibodies and may offer more significant benefits than other intracellular and periplasmic proteins that have been previously studied. Omp25's acceptable molecular weight (about 25 kDa) is also promising for excellent expression and an easy subsequent purification process. We hypothesized that the co‐administration of immunomodulatory cytokines and antigens would boost the immunogenicity of the fusion protein (romp25–Usp45) and the efficacy of our constructed vaccine when Usp45 was expressed as an omp25 secretion factor of *B. abortus* in the future in vivo study on the topic of vaccination against brucellosis. This is the first research on the cloning, expression and purification of the romp25/Usp45 fusion protein. Prior research was conducted on the co‐expression of secretion factor and antigen. Previous studies have shown that mice inoculated with bacterial species that simultaneously generated tetanus toxin fragment C (TTFC), IL‐2 or IL‐6 had more significant antibody titres for test TTFC (Gupta et al., 2020).

To test the notion, however, a different experiment that compares the immunity of omp16, whether fused or not, to IL‐2 and performs in vivo study is needed. This study is the first to use a unique promoter to produce the recombinant omp25 polypeptide in *L. lactis*. Low pH‐induced expression when cells were grown on glucose shift from the post‐exponential to stationary phases (Zeng et al., 2022; Azadbakht et al., 2022). Without the use of costly or hazardous exogenous compounds and the ability to recover the protein quickly in subsequent steps, this induction is beneficial as an alternative and cost‐effective tool for the production of heterologous proteins of therapeutic or technological interest. Some of the issues stem from synthesizing proteins using a pH‐inducible promoter controlled by the extracellular level of ions like Cl or Zn (Peter et al., 2022). Consequently, expression optimization should be carried out to enable high‐yield manufacturing. It is essential to highlight that while though *B. abortus* recombinant food grad vaccines have highly positive outcomes in mouse models; the protective immunity shown in these models could not accurately represent the level of protection attained in wild hosts like cattle following immunization (Corner et al., 2010; Kargar et al., 2014). Before administering to cattle, more research evaluating protective effects in animal studies such as rats, guinea pigs and monkeys is thus urged. Recombinant vaccines are economically inappropriate for immunizing cattle because they require numerous booster injections, adjuvants and a mix of various antigens (Dorneles, Sriranganathan, et al., 2015; Dorneles, Teixeira‐Carvalho, et al., 2015). Therefore, to make these vaccines inexpensive for widespread use, it is necessary to lower the cost of manufacturing, look for efficient and affordable adjuvants and reduce the cost of recombinant protein purification.

## 5 CONCLUSIONS

As a result, our study offers a novel method for using the food‐grade, non‐pathogenic and noncommercial bacterium *L. lactis* as a protein cell factory to produce the novel immunogenic fusion candidate romp25. This method offers an appealing new approach to assessing the cost‐effective, safe, sustainable, simple pilot development of pharmaceutical products. It suggests a workable method for delivering additional heterologous protein antigens. We have already started doing additional studies in our labs, such as testing the immunogenicity and protective effectiveness of this recombinant *L. lactis*–romp25 strain as a potential oral vaccine candidate vs. virulent *Brucella* infection challenge in mice.

## AUTHOR CONTRIBUTIONS

Somaye Tirbakhsh Gouran conducted research and drafted the manuscript; Abbas Doosti conceived and designed research and made manuscript revision; Mohammad Saeid Jami designed research and analysed data; Somaye Tirbakhsh Gouran provided funding support. All authors read and approved the final manuscript.

## CONFLICT OF INTEREST STATEMENT

The authors declare that they have no conflict of interest.

## ETHICS STATEMENT

All animal protocols were carried out following the regulations established by the Institutional Animal Care and Use Committee of Islamic Azad University, Shahrekord, Iran (IR.IAU.SHK.REC.1401.001).

### PEER REVIEW

The peer review history for this article is available at https://publons.com/publon/10.1002/vms3.1173.

## Data Availability

The data generated or analysed during this study are included in this article and its additional materials.

## References

[vms31173-bib-0001] Azadbakht, N. , Doosti, A. , & Jami, M. S. (2022). CRISPR/Cas9‐mediated LINC00511 knockout strategies, increased apoptosis of breast cancer cells via suppressing antiapoptotic genes. Biological Procedures Online, 24(1), 8. 35790898 10.1186/s12575-022-00171-1 PMC9254607

[vms31173-bib-0002] Beiranvand, S. , Piri‐Gharaghie, T. , Dehganzad, B. , Khedmati, F. , Jalali, F. , AsadAlizadeh, M. , & Momtaz, H. (2022). Novel NAD‐independent *Avibacterium paragallinarum*: Isolation, characterization and molecular identification in Iran. Veterinary Medicine and Science, 8(3), 1157–1165. 35182464 10.1002/vms3.754 PMC9122455

[vms31173-bib-0003] Bermúdez‐Humarán, L. G. , Kharrat, P. , Chatel, J. M. , & Langella, P. (2011). Lactococci and lactobacilli as mucosal delivery vectors for therapeutic proteins and DNA vaccines. Microbial Cell Factories, 10(1), S4. 21995317 10.1186/1475-2859-10-S1-S4 PMC3231930

[vms31173-bib-0004] Bermúdez‐Humarán, L. G. , Langella, P. , Cortes‐Perez, N. G. , Gruss, A. , Tamez‐Guerra, R. S. , Oliveira, S. C. , Cardenas, O. S. , Montes de Oca‐Luna, R. , & Le Loir, Y. (2003). Intranasal immunization with recombinant *Lactococcus lactis* secreting murine interleukin‐12 enhances antigen‐specific Th1 cytokine production. Infection and Immunity, 71(4), 1887–1896. 12654805 10.1128/IAI.71.4.1887-1896.2003 PMC152106

[vms31173-bib-0005] Corner, L. A. , Costello, E. , O'Meara, D. , Lesellier, S. , Aldwell, F. E. , Singh, M. , Hewinson, R. G. , Chambers, M. A. , & Gormley, E. (2010). Oral vaccination of badgers (*Meles meles*) with BCG and protective immunity against endobronchial challenge with *Mycobacterium bovis* . Vaccine, 28(38), 6265–6272. 20637774 10.1016/j.vaccine.2010.06.120

[vms31173-bib-0006] Daniel, C. , Poiret, S. , Dennin, V. , Boutillier, D. , & Pot, B. (2013). Bioluminescence imaging study of spatial and temporal persistence of *Lactobacillus plantarum* and *Lactococcus lactis* in living mice. Applied and Environmental Microbiology, 79(4), 1086–1094. 23204409 10.1128/AEM.03221-12 PMC3568624

[vms31173-bib-0007] Díaz‐Dinamarca, D. A. , Salazar, M. L. , Castillo, B. N. , Manubens, A. , Vasquez, A. E. , Salazar, F. , & Becker, M. I. (2022). Protein‐based adjuvants for vaccines as immunomodulators of the innate and adaptive immune response: Current knowledge, challenges, and future opportunities. Pharmaceutics, 14(8), 1671. 36015297 10.3390/pharmaceutics14081671 PMC9414397

[vms31173-bib-0008] Dadar, M. , Tiwari, R. , Sharun, K. , & Dhama, K. (2021). Importance of brucellosis control programs of livestock on the improvement of one health. Veterinary Quarterly, 41(1), 137–151. 33618618 10.1080/01652176.2021.1894501 PMC7946044

[vms31173-bib-0009] Kargar, M. , Mohammadalipour, Z. , Doosti, A. , Lorzadeh, S. , & Japoni‐Nejad, A. (2014). High prevalence of class 1 to 3 integrons among multidrug‐resistant diarrheagenic *Escherichia coli* in southwest of Iran. Osong Public Health and Research Perspectives, 5(4), 193–198. 25379369 10.1016/j.phrp.2014.06.003 PMC4215003

[vms31173-bib-0010] Dorneles, E. , Sriranganathan, N. , & Lage, A. P. (2015). Recent advances in *Brucella abortus* vaccines. Veterinary Research, 46(1), 76. 26155935 10.1186/s13567-015-0199-7 PMC4495609

[vms31173-bib-0011] Dorneles, E. M. , Teixeira‐Carvalho, A. , Araújo, M. S. , Sriranganathan, N. , & Lage, A. P. (2015). Immune response triggered by *Brucella abortus* following infection or vaccination. Vaccine, 33(31), 3659–3666. 26048781 10.1016/j.vaccine.2015.05.057

[vms31173-bib-0012] Franc, K. A. , Krecek, R. C. , Häsler, B. N. , & Arenas‐Gamboa, A. M. (2018). Brucellosis remains a neglected disease in the developing world: A call for interdisciplinary action. BMC Public Health, 18(1), 125. 29325516 10.1186/s12889-017-5016-y PMC5765637

[vms31173-bib-0013] Guillot, A. , Boulay, M. , Chambellon, E. , Gitton, C. , Monnet, V. , & Juillard, V. (2016). Mass spectrometry analysis of the extracellular peptidome of *Lactococcus lactis*: Lines of evidence for the coexistence of extracellular protein hydrolysis and intracellular peptide excretion. Journal of Proteome Research, 15(9), 3214–3224. 27439475 10.1021/acs.jproteome.6b00424

[vms31173-bib-0014] Gupta, S. , Mohan, S. , Somani, V. K. , Aggarwal, S. , & Bhatnagar, R. (2020). Simultaneous immunization with omp25 and L7/L12 provides protection against brucellosis in mice. Pathogens, 9(2), 152. 32102449 10.3390/pathogens9020152 PMC7175130

[vms31173-bib-0015] Ghajari, G. , Nabiuni, M. , & Amini, E. (2021). The association between testicular toxicity induced by Li2Co3 and protective effect of *Ganoderma lucidum*: Alteration of Bax & c‐Kit genes expression. Tissue and Cell, 72, 101552. 33992978 10.1016/j.tice.2021.101552

[vms31173-bib-0016] Hosseini, S. M. , Taheri, M. , Nouri, F. , Farmani, A. , Moez, N. M. , & Arabestani, M. R. (2022). Nano drug delivery in intracellular bacterial infection treatments. Biomedicine & Pharmacotherapy, 146, 112609. 35062073 10.1016/j.biopha.2021.112609

[vms31173-bib-0017] Heidary, M. , Dashtbin, S. , Ghanavati, R. , Ari, M. M. , Bostanghadiri, N. , Darbandi, A. , Navidifar, T. , & Talebi, M. (2022). Evaluation of brucellosis vaccines: A comprehensive review. Frontiers in Veterinary Science, 9, 925773. 35923818 10.3389/fvets.2022.925773 PMC9339783

[vms31173-bib-0018] He, S. , Gong, F. , Guo, Y. , & Zhang, D. (2012). Food‐grade selection markers in lactic acid bacteria. TAF Preventive Medicine Bulletin, 11(1), 499.

[vms31173-bib-0019] Jawan, R. , Abbasiliasi, S. , Mustafa, S. , Kapri, M. R. , Halim, M. , & Ariff, A. B. (2021). In vitro evaluation of potential probiotic strain *Lactococcus lactis* Gh1 and its bacteriocin‐like inhibitory substances for potential use in the food industry. Probiotics and Antimicrobial Proteins, 13(2), 422–440. 32728855 10.1007/s12602-020-09690-3

[vms31173-bib-0020] Jamil, T. , Khan, A. U. , Saqib, M. , Hussain, M. H. , Melzer, F. , Rehman, A. , Shabbir, M. Z. , Khan, M. A. , Ali, S. , Shahzad, A. , Khan, I. , Iqbal, M. , Ullah, Q. , Ahmad, W. , Mansoor, M. K. , Neubauer, H. , & Schwarz, S. (2021). Animal and human brucellosis in Pakistan. Frontiers in Public Health, 9, 660508. 34395357 10.3389/fpubh.2021.660508 PMC8362930

[vms31173-bib-0021] Levit, R. , Cortes‐Perez, N. G. , de Moreno de Leblanc, A. , Loiseau, J. , Aucouturier, A. , Langella, P. , LeBlanc, J. G. , & Bermúdez‐Humarán, L. G. (2022). Use of genetically modified lactic acid bacteria and bifidobacteria as live delivery vectors for human and animal health. Gut Microbes, 14(1), 2110821. 35960855 10.1080/19490976.2022.2110821 PMC9377234

[vms31173-bib-0022] Li, Z. , Wang, S. , Wei, S. , Yang, G. , Zhang, C. , Xi, L. , Zhang, J. , Cui, Y. , Hao, J. , Zhang, H. , & Zhang, H. (2022). Immunization with a combination of recombinant *Brucella abortus* proteins induces T helper immune response and confers protection against wild‐type challenge in BALB/c mice. Microbial Biotechnology, 15(6), 1811–1823. 35166028 10.1111/1751-7915.14015 PMC9151338

[vms31173-bib-0023] Le Loir, Y. , Azevedo, V. , Oliveira, S. C. , Freitas, D. A. , Miyoshi, A. , Bermúdez‐Humarán, L. G. , Nouaille, S. , Ribeiro, L. A. , Leclercq, S. , Gabriel, J. E. , Guimaraes, V. D. , Oliveira, M. N. , Charlier, C. , Gautier, M. , & Langella, P. (2005). Protein secretion in *Lactococcus lactis*: An efficient way to increase the overall heterologous protein production. Microbial Cell Factories, 4(1), 2. 15631634 10.1186/1475-2859-4-2 PMC545053

[vms31173-bib-0024] Pizarro‐Cerdá, J. , Moreno, E. , & Gorvel, J. P. (2000). Invasion and intracellular trafficking of *Brucella abortus* in nonphagocytic cells. Microbes and Infection, 2(7), 829–835. 10955964 10.1016/s1286-4579(00)90368-x

[vms31173-bib-0025] Piri‐Gharaghie, T. , Beiranvand, S. , Riahi, A. , Shirin, N. J. , Badmasti, F. , Mirzaie, A. , Elahianfar, Y. , Ghahari, S. , Ghahari, S. , Pasban, K. , & Hajrasouliha, S. (2022). Fabrication and characterization of thymol‐loaded chitosan nanogels: Improved antibacterial and anti‐biofilm activities with negligible cytotoxicity. Chemistry & Biodiversity, 19(3), e202100426. 34989129 10.1002/cbdv.202100426

[vms31173-bib-0026] Plavec, T. V. , Ključevšek, T. , & Berlec, A. (2021). Introduction of modified BglBrick system in *Lactococcus lactis* for straightforward assembly of multiple gene cassettes. Frontiers in Bioengineering and Biotechnology, 9, 797521. 34957084 10.3389/fbioe.2021.797521 PMC8703077

[vms31173-bib-0027] Piri‐Gharaghie, T. , Doosti, A. , & Mirzaei, S. A. (2022). Identification of antigenic properties of *Acinetobacter baumannii* proteins as novel putative vaccine candidates using reverse vaccinology approach. Applied Biochemistry and Biotechnology, 194(10), 4892–4914. 35670904 10.1007/s12010-022-03995-5

[vms31173-bib-0028] Piri‐Gharaghie, T. , Doosti, A. , & Mirzaei, S. A. (2023). Novel adjuvant nano‐vaccine induced immune response against *Acinetobacter baumannii* . AMB Express, 13(1), 31. 36905472 10.1186/s13568-023-01531-0 PMC10008545

[vms31173-bib-0029] Piri‐Gharaghie, T. , Ghajari, G. , Hassanpoor, M. , Jegargoshe‐Shirin, N. , Soosanirad, M. , Khayati, S. , Farhadi‐Biregani, A. , & Mirzaei, A. (2023). Investigation of antibacterial and anticancer effects of novel niosomal formulated Persian Gulf Sea cucumber extracts. Heliyon, 9(3), e14149. 10.1016/j.heliyon.2023.e14149 36938478 PMC10018472

[vms31173-bib-0030] Peter, S. B. , Qiao, Z. , Godspower, H. N. , Ajeje, S. B. , Xu, M. , Zhang, X. , Yang, T. , & Rao, Z. (2022). Biotechnological innovations and therapeutic application of *Pediococcus* and lactic acid bacteria: The next‐generation microorganism. Frontiers in Bioengineering and Biotechnology, 9, 802031. 35237589 10.3389/fbioe.2021.802031 PMC8883390

[vms31173-bib-0031] Reschner, A. , Hubert, P. , Delvenne, P. , Boniver, J. , & Jacobs, N. (2008). Innate lymphocyte and dendritic cell cross‐talk: A key factor in the regulation of the immune response. Clinical & Experimental Immunology, 152(2), 219–226. 18336590 10.1111/j.1365-2249.2008.03624.x PMC2384094

[vms31173-bib-0032] Ribeiro, L. A. , Azevedo, V. , Le Loir, Y. , Oliveira, S. C. , Dieye, Y. , Piard, J. C. , Gruss, A. , & Langella, P. (2002). Production and targeting of the *Brucella abortus* antigen L7/L12 in *Lactococcus lactis*: A first step towards food‐grade live vaccines against brucellosis. Applied and Environmental Microbiology, 68(2), 910–916. 11823235 10.1128/AEM.68.2.910-916.2002 PMC126665

[vms31173-bib-0033] Rezaei, M. , Rabbani Khorasgani, M. , Zarkesh Esfahani, S. H. , Emamzadeh, R. , & Abtahi, H. (2020). Production of *Brucella melitensis* omp16 protein fused to the human interleukin 2 in *Lactococcus lactis* MG1363 toward developing a *Lactococcus*‐based vaccine against brucellosis. Canadian Journal of Microbiology, 66(1), 39–45. 31574230 10.1139/cjm-2019-0261

[vms31173-bib-0034] Saxena, N. , Singh, B. B. , & Saxena, H. M. (2018). Brucellosis in sheep and goats and its serodiagnosis and epidemiology. International Journal of Current Microbiology and Applied Sciences, 7(1), 1848–1877.

[vms31173-bib-0035] Sáez, D. , Fernández, P. , Rivera, A. , Andrews, E. , & Oñate, A. (2012). Oral immunization of mice with recombinant *Lactococcus lactis* expressing Cu, Zn superoxide dismutase of *Brucella abortus* triggers protective immunity. Vaccine, 30(7), 1283–1290. 22222868 10.1016/j.vaccine.2011.12.088

[vms31173-bib-0036] Singh, N. , Mishra, T. , Singh, K. , & Singh, J. (2017). Microbial and non‐microbial pyrogens in healthcare products: Risks, quality control and regulatory aspects. Applied Clinical Research, Clinical Trials and Regulatory Affairs, 4(1), 4–15.

[vms31173-bib-0037] Taghinezhad, S. , Mohseni, A. H. , Bermúdez‐Humarán, L. G. , Casolaro, V. , Cortes‐Perez, N. G. , Keyvani, H. , & Simal‐Gandara, J. (2021). Probiotic‐based vaccines may provide effective protection against COVID‐19 acute respiratory disease. Vaccines, 9(5), 466. 34066443 10.3390/vaccines9050466 PMC8148110

[vms31173-bib-0038] Zhang, Y. , Wang, X. , Li, Z. , Zhang, J. , Wang, Y. , Wu, C. , Chen, C. , Li, J. , & Zhang, H. (2022). *Brucella melitensis* outer membrane protein 25 interacts with ferritin heavy polypeptide 1 in human trophoblast cells. Molecular Medicine Reports, 26(1), 224. 35593274 10.3892/mmr.2022.12740

[vms31173-bib-0039] Zeng, L. , Walker, A. R. , Burne, R. A. , & Taylor, Z. A. (2022). Glucose phosphotransferase system modulates pyruvate metabolism, bacterial fitness, and microbial ecology in oral streptococci. Journal of Bacteriology, 205(1), e0035222. 36468868 10.1128/jb.00352-22 PMC9879115

[vms31173-bib-0040] Zeinali, T. , Faraji, N. , Joukar, F. , Mirzaei, M. K. , Jalali, H. K. , Shenagari, M. , & Mansour‐Ghanaei, F. (2022). Gut bacteria, bacteriophages, and probiotics: Tripartite mutualism to quench the SARS‐CoV2 storm. Microbial Pathogenesis, 170, 105704. 35948266 10.1016/j.micpath.2022.105704 PMC9357283

